# Associations of metabolic score for insulin resistance (METS-IR) with cardiovascular mortality risk in peritoneal dialysis patients

**DOI:** 10.1371/journal.pone.0349680

**Published:** 2026-11-11

**Authors:** Chun-Chieh Tsai, Yao-Peng Hsieh, Yu-Jun Chang, Shr-Mei Tsai, Ping-Fang Chiu

**Affiliations:** 1 Division of Nephrology, Department of Internal Medicine, Changhua Christian Hospital, Changhua, Taiwan; 2 Big Data Center, Changhua Christian Hospital, Changhua, Taiwan; 3 Department of Nursing, Changhua Christian Hospital, Changhua, Taiwan; Tehran University of Medical Sciences, IRAN, ISLAMIC REPUBLIC OF

## Abstract

**Background:**

Metabolic score for insulin resistance (METS-IR) is considered a simple, reliable, and cost-effective alternative indicator of insulin resistance (IR). Our study aimed to assess whether the METS-IR index was associated with an increased risk of cardiovascular (CV) death in our peritoneal dialysis (PD) cohort.

**Methods:**

This observational study included 553 patients commencing PD for end-stage renal disease between 2003 and 2017, and they were divided into three groups according to the tertiles of METS-IR. The assessed predictor was the METS-IR index at enrollment. Cox proportional hazards models and competing risk analysis using the Fine-Gray models were used to analyze the association between the METS-IR index and CV mortality, with adjustments of potential confounding factors including medication use, demographic characteristics, comorbidities, PD-related data, and laboratory data. Furthermore, receiver operating characteristic (ROC) curve analysis was used to determine the exploratory cutoff value of the METS-IR index for predicting CV mortality, and the area under the ROC curve (AUC) was calculated.

**Results:**

During follow-up, the crude CV mortality rate increased significantly, with 16 CV deaths (8.6%) in the first tertile of the METS-IR index, 32 CV deaths (17.4%) in the second tertile, and 41 CV deaths (22.3%) in the third tertile (p < 0.001). In the full adjusted model, patients in tertile 2 were associated with an HR of 1.90 (95% CI, 1.01–3.61) and those in tertile 3 was associated with an HR of 1.91 (95% CI, 1.03–3.55) compared with tertile 1. The exploratory cut-off point of METS-IR index was 33.3 (75.3% sensitivity and 48.3% specificity) with an AUC (95% CI) of 0.617 (0.557–0.677; p < 0.001), indicating modest discrimination. The results from the Fine-Gray model are consistent with those from the Cox model.

**Conclusions:**

METS-IR can identify PD patients at high risk of CV death. However, METS-IR has limited discriminative ability in predicting CV mortality and further research is needed to validate its potential role in risk stratification.

## Introduction

Peritoneal dialysis (PD) is one of the mainstays of long-term available renal replacement therapy for patients with end-stage renal disease (ESRD) [1]. The utilization of PD varies a lot around the world, ranging from almost zero to over 50%. Since 2006, PD use has increased in several countries, including Chile, Spain, Taiwan, Thailand, and the United States [2]. PD is most widely used in Hong Kong with a prevalence rate of 69% [3]. Despite recent advances in dialysis, ESRD patients experience poor quality of life, high hospitalization rates, and significant mortality, primarily attributed to cardiovascular (CV) causes [4]. Multiple factors may affect the risk of death in PD patients. Commonly used nutritional indicators, such as serum levels of albumin, potassium, and body mass index (BMI), were reported to have poor predictive value for shortened life expectancy in PD patients [5].

Insulin resistance (IR) is a state of an impaired biological response to insulin actions in the body and plays a key role in the disorder of lipid and glucose metabolism [6]. The prevalence of IR has been on the rise worldwide and is considered a crucial factor contributing cardiovascular diseases (CVD) by promoting coronary atherosclerosis and inducing myocardial damage [7]. The gold standard method for quantifying insulin sensitivity, the hyperinsulinemic euglycemic clamp, is not suitable for clinical research and practice due to its cost and complexity. The recently developed metabolic score for insulin resistance (METS-IR) index combines fasting blood glucose (FBG), triglycerides (TG), high-density lipoprotein (HDL-C), and body mass index (BMI). It has better concordance with the hyperinsulinemic-euglycemic clamp technique and is intended to be a practical, reliable and accessible alternative estimate to IR [8].

A number of studies have found METS-IR to be strongly correlated with various cardiometabolic disorders, such as hypertension, type 2 diabetes mellitus (DM) and incident ischemic heart disease [9–11]. Currently, there are few studies on the relationship between METS-IR and CV mortality, especially in PD patients. Therefore, this study aims to explore the relationship between METS-IR index and CV mortality in PD patients.

## Materials and methods

### Participants and measurements

A retrospective study in a PD cohort was conducted between January 2003 and June 2017 at the department of Nephrology, Changhua Christian Hospital, Taiwan. Patients who commenced PD treatment for at least three months and aged over 18 years were enrolled in this study. All study procedures were approved by the Institutional Review Board of Changhua Christian Hospital (CCH IRB No. 241116) and performed in accordance with the principles of Declaration of Helsinki. All patients were followed up until death or administrative censoring, which included switching to hemodialysis, receipt of a kidney transplant, or the end of the study period, whichever occurred first. No patient was lost to follow-up. All the patients’ clinical medical records and information were de-identified and anonymized before analysis. We were granted an exemption from getting written informed consents from each participant because of a retrospective study reviewing anonymized medical records.

### Data collection and definitions

Data were retrieved from well-established electronic medical records on March 25, 2025 and included sociodemographic characteristics, anthropometric data, comorbidities, laboratory tests, medications, and PD-related parameters. As per protocol, our PD patients were followed up monthly in the outpatient clinic and had their blood tests checked routinely. Demographic characteristics consisted of age and gender. Body mass index (BMI) was calculated as weight (kg) divided by squared height (m^2^). Hematological and biochemical tests included serum creatinine, albumin, white blood cell (WBC) count, hemoglobin, glycated hemoglobin (HbA1C), fasting blood glucose (FBG), total cholesterol (TC), triglyceride (TG), low-density lipoprotein cholesterol (LDL-C), and high-density lipoprotein cholesterol (HDL-C), calcium, and phosphate. Pharmacotherapy with lipid- lowering drugs and antihypertensive agents (renin-angiotensin-aldosterone system inhibitor, RAAS inhibitor) was also recorded. Renal twenty-four-hour urea and creatinine clearances were averaged to assess residual renal function (RRF). Dialysis adequacy assessment include weekly total urea clearance (Kt/V). Baseline comorbidities included diabetes mellitus (DM), hypertension and CVD.

### Exposure Definition

METS-IR was calculated as the Ln [(2 × FPG (mg/dL) + fasting TG (mg/dL) × BMI (kg/m^2^)]/ Ln [(HDL-C (mg/dL)]. The study cohort was divided into three groups based on the tertiles of the METS-IR index: the first tertile (METS-IR index < 31.3), the second tertile (31.3 ≤ METS-IR index ≤ 39.0), and the third tertile (METS-IR index > 39.0).

### Study outcomes

The primary study endpoint was CV mortality which was defined as death from coronary heart disease, heart failure, myocardial infarction, arrhythmia, cardiomyopathy, or cerebrovascular disorder.

### Statistical analysis

The METS-IR index was used to divide the whole study cohort into three groups (tertiles) to investigate the associations of METS-IR index with clinical outcomes. Continuous variables are expressed as mean and standard deviation (SD) or median and interquartile range (IQR), while categorical variables are expressed as frequency and percentage. We used METS-IR as an independent variable and stratified the study population characteristics and clinical indexes according to their tertiles. We then compared the baseline characteristics of the groups using Mann-Whitney U test for continuous variables and Chi-square test for categorical variables.

Kaplan-Meier curves were plotted to analyze survival time among the METS-IR tertiles, and the survival differences was compared using the log-rank test. The Cox proportional hazards regression model was used to estimate the hazard ratio (HR) and its corresponding 95% confidence interval (CI) to examine the relationship between METS-IR index and CV mortality. The proportional hazards assumption for the Cox models was assessed using Schoenfeld residual-based tests, and no material violation was identified in the models. Since non-CV cardiovascular deaths may constitute competing events, a competing risk analysis was also performed using the Fine-Gray subdistribution hazards model to estimate the subdistributed hazard ratio (sHR).

We performed stepwise model adjustments to test the independent association between the METS-IR index and our study outcome. Crude model represented a univariate Cox regression analysis without adjustments. Model 1 was adjusted for age and sex. Model 2 was adjusted for variables in Model 1 plus medications use and comorbid conditions. Model 3 was adjusted for variables in Model 2 plus PD-related parameters and laboratory data. To mitigate the effects of collinearity, which further complicates the interpretation of independence results, HDL-C, BMI, TG, fasting blood glucose, and HbA1C were not adjusted, as they are index components of METS-IR.

There is no normal reference range for the METS-IR index, nor is there an optimal cut-off value. Sensitivity (SS) and specificity (SP) are defined as the probability of correctly identifying a subject with the disease and the probability of accurately identifying a subject without the disease, respectively. The exploratory (optimal) cutoff value of METS-IR for predicting CV mortality was determined using the maximum value of Youden index = SS + SP − 1, and the area under the receiver operating characteristic (ROC) curve (AUC) was calculated accordingly. We examined the independent role of the METS-IR in predicting CV mortality when using the exploratory METS-IR index through ROC analysis. Furthermore, we analyzed METS-IR as a continuous variable, including per 1-SD and per 10-unit increase. All statistical analyses were performed using IBM SPSS Statistics for Windows, version 22.0 (IBM Corp., Armonk, NY) and R version 4.1.2. Two-sided p-values ≤ 0.05 were considered statistically significant.

## Results

### Patients’ baseline characteristics

Baseline characteristics of the eligible ESRD patients on PD (n = 553; 265 men and 288 women) according to the METS-IR tertiles were shown in Table 1. The BMI and mean age of the study population were 23.5 ± 3.9 kg/m2 and 52.5 ± 15.2 years, respectively. The mean FPG concentration was 116.9 ± 53.3 mg/dL, the mean triglycerides level was 135.6 ± 83.2 mg/dL, the mean HDL level was 51.9 ± 17 mg/dL, and the mean METS-IR index value was 35.9 ± 8.5. The patient’s age increased significantly from METS-IR tertile 1 group through tertile 3 (p < 0.001). The higher METS-IR index groups had a significantly higher prevalence of DM and CVD (both p values < 0.001). Regarding PD- related parameters, there were significant differences in weekly total Kt/V urea and residual renal function among the three groups.

**Table 1:** Baseline characteristics of the study population by the METS-IR groups.

	METS-IR tertile 1	METS-IR tertile 2	METS-IR tertile 3	*p*-Value
	(METS-IR < 31.3)	(31.3 ≤ METS-IR ≦ 39.0)	(METS-IR > 39.0)	
**Number of Patients**	n = 185	n = 184	n = 184	--
Sex, men	60 (32.4%)	99 (53.8%)	106 (57.6%)	<0.001
Age (years)	48.4 ± 16.9	53.0 ± 13.5	56.1 ± 13.9	<0.001
Body mass index (kg/m^2^)	19.9 ± 2.0	23.3 ± 2.0	27.4 ± 3.1	<0.001
**Comorbidity Disease**				
Hypertension	160 (86.5%)	158 (85.9%)	164 (89.1%)	0.610
Diabetes mellitus	23 (12.4%)	56 (30.4%)	111 (60.3%)	<0.001
Cardiovascular disease	47 (25.4%)	59 (32.1%)	85 (46.2%)	<0.001
**Medication use**				
RAAS inhibitor	108 (58.4%)	118 (64.1%)	127 (69.0%)	0.103
Lipid-lowering agents	22 (11.9%)	43 (23.4%)	65 (35.3%)	<0.001
**Laboratory data**				
Albumin (g/dL)	3.4 (3.0–3.8)	3.4 (3.0–3.8)	3.2 (2.9–3.6)	<0.001
Fasting blood glucose (mg/dL)	98.5 (90-115.5)	118.5 (97.5-168)	99 (90-120)	<0.001
Creatinine (mg/dL)	9.7 (8.2–12.1)	9.6 (8.1–11.9)	9.6 (8.1–11.8)	0.669
Calcium (mg/dL)	8.3 (7.9–8.9)	8.3 (8.0–8.8)	8.3 (7.9–8.8)	0.947
Phosphate (mg/dL)	5.4 (4.7–6.4)	5.5 (4.7–6.4)	5.5 (4.6–6.4)	0.717
Cholesterol (mg/dL)	181.5 (151–222.5)	187.5 (154.5–233.5)	187.0 (155.0–232.0)	0.179
HDL–C (mg/dL)	50.0 (42.0–58.0)	38.5 (32.0–45.0)	50.0 (39.0–62.0)	<0.001
LDL–C (mg/dL)	104.8 (80.7–136.3)	113.8 (85.9–138.0)	111.0 (85.8–138.0)	0.472
Triglyceride (mg/dL)	110.0 (78.0–159.5)	153.0 (104.0–213.5)	115.0 (83.0–165.0)	<0.001
Hemoglobin (g/dL)	8.7 (7.6–9.6)	8.7 (7.9–9.5)	8.7 (7.7–9.5)	0.484
HbA1C (%)	5.5 (4.9–6.4)	6.0 (5.3–6.9)	5.5 (5.0–6.3)	<0.001
WBC (× 10^3^/μL)	6.7 (5.0–8.6)	7.4 (6.0–9.4)	6.9 (5.3–8.7)	<0.001
**PD-related parameters**				
Weekly total Kt/V urea	2.0 (1.7–2.3)	1.8 (1.6–2.1)	2.0 (1.7–2.3)	<0.001
Residual renal function (mL/min/1.73 m^2^)	3.0 (2.3–4.1)	3.0 (2.2–4.5)	3.0 (2.1–4.0)	0.001

Values are expressed as mean ± SD, median (interquartile range) or number (percentage).

Abbreviations: RAAS inhibitor, renin-angiotensin-aldosterone system inhibitor; BMI, body mass index; WBC, white blood cell count; nPNA, normalized protein nitrogen appearance; HDL-C: high density lipoprotein- cholesterol; LDL-C: low density lipoprotein- cholesterol.

### Association of METS-IR index (tertiles) with CV mortality

During a median follow-up of 35.7 months (range 3.0–195.2 months), 142 patients died while 89 patients died from CVD. The crude CV mortality rate increased significantly, with 16 CV deaths (8.6%) in the first tertile of the METS-IR index, 32 CV deaths (17.4%) in the second tertile, and 41 CV deaths (22.3%) in the third tertile (p < 0.001). Kaplan-Meier survival curves showed that there was a significant difference in the incidence of CV mortality (Fig 1; p < 0.001). The results of crude and multiple-step adjusted models of Cox proportional hazards regression analyses were listed in Table 2. In the unadjusted model, compared with the first METS-IR index tertile, the HRs of CV mortality for the second and third tertiles increased in a dose-dependent manner (HR 2.32 95% CI, 1.27–4.23 for tertile 2; HR 3.56 95% CI, 1.99–6.37 for tertile 3). After multivariable adjustments (model 3), patients in the METS-IR tertile 2 were associated with an HR of 1.90 (95% CI, 1.01–3.61) and those in the METS-IR tertile 3 was associated with an HR of 1.91 (95% CI, 1.03–3.55) compared with METS-IR tertile 1.

**Table 2:** Cox and Fine–Gray regression models of CV mortality for METS-IR groups.

METS-IR category: ETS-IR tertile 1 group: METS-IR < 31.3 METS-IR tertile 2 group: 31.3 ≦ METS-IR ≦ 39.0 METS-IR tertile 3 group: METS-IR > 39.0		Cox regression models		Fine-Gray models	
		HR (95% CI)	p-value	sHR (95% CI)	*P*-value
**(A)**Crude model	Tertile 1	reference		reference	
	Tertile 2	2.32(1.27-4.23)	0.006	2.30 (1.26-4.19)	0.006
	Tertile 3	3.56(1.99-6.37)	<0.001	3.52 (1.99-6.22)	<0.001
Model 1	Tertile 1	reference		reference	
	Tertile 2	2.37(1.29-4.35)	0.006	2.37 (1.27-4.39)	0.006
	Tertile 3	3.04(1.69-5.45)	<0.001	3.21 (1.78-5.81)	<0.001
Model 2	Tertile 1	reference		reference	
	Tertile 2	1.87(1.01-3.47)	0.048	2.03 (1.09-3.76)	0.025
	Tertile 3	2.09(1.13-3.87)	0.018	2.33 (1.25-4.35)	0.008
Model 3	Tertile 1	reference		reference	
	Tertile 2	1.90(1.01-3.61)	0.047	2.22 (1.12-4.38)	0.022
	Tertile 3	1.91(1.03-3.55)	0.040	2.32 (1.19-4.51)	0.013
***(B) sensitivity tests***					
ROC-derived cutoff (Model 3)	METS-IR < 33.3	reference		reference	
	METS-IR ≧ 33.3	1.89(1.12-3.23)	0.018	2.27 (1.30–3.96)	0.004

Crude model: unadjusted hazard ratios (HRs) and subdistribution hazard ratios (sHRs) for METS-IR categories. HRs were estimated using Cox proportional hazards models, and sHRs were estimated using Fine–Gray subdistribution hazard models.

Model 1: METS-IR, age and sex.

Model 2: Model 1 plus medications and comorbidities.

Model 3: Model 2 plus PD-related parameters and laboratory data.

To mitigate the effects of collinearity, which further complicates the interpretation of independence results, HDL-C, BMI, TG, fasting blood glucose, and HbA1C were not adjusted,

Abbreviations: METS-IR, metabolic score for insulin resistance; CV, cardiovascular; HR, hazard ratio; sHR, subdistribution hazard ratio; CI, confidence interval.

**Fig 1:**
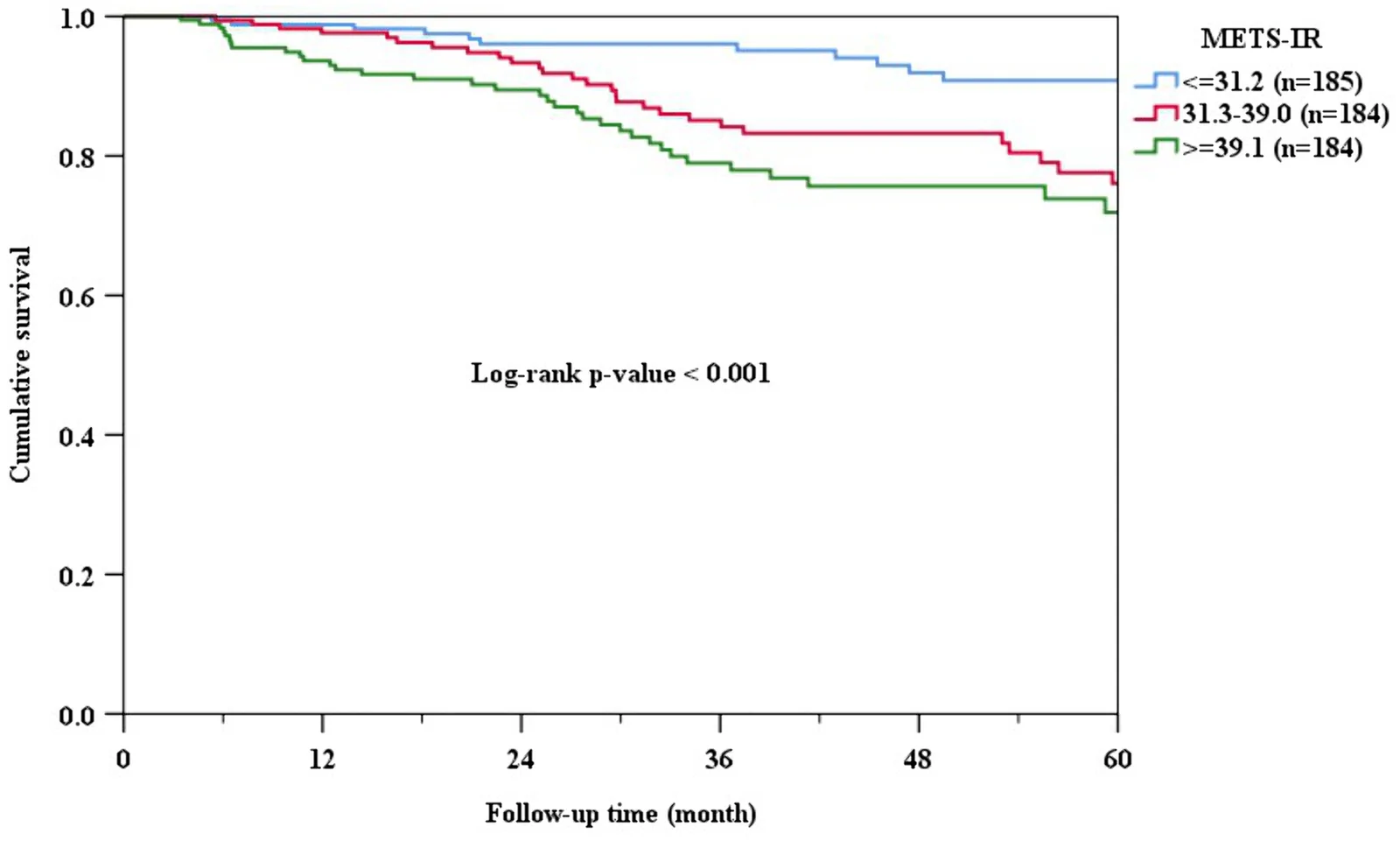
Kaplan-Meier curve of cardiovascular survival across METS-IR tertile groups (log-rank test, p < 0.001).

### Association of METS-IR index as a continuous variable with CV mortality

When METS-IR was analyzed as a continuous variable (per 1-SD increase and per 10-unit increase), higher METS-IR values were associated with an increased risk of CV mortality. Consistent results were observed both in the Cox regression models and Fine-Gray models (Table 3).

**Table 3:** Cox and Fine–Gray regression analyses of cardiovascular mortality with METS-IR modeled as a continuous variable.

	Cox regression models		Fine-Gray models	
	HR (95% CI)	*P*-value	sHR (95% CI)	*P*-value
**(A) Per 1-SD increase**				
Crude model	1.53 (1.26–1.86)	<0.001	1.50 (1.24–1.80)	<0.001
Model 1	1.45 (1.19–1.77)	<0.001	1.46 (1.20–1.78)	<0.001
Model 2	1.26 (1.00–1.57)	0.046	1.29 (1.03–1.60)	0.024
Model 3	1.26 (1.00–1.60)	0.055	1.33 (1.05–1.68)	0.017
**(B) Per 10-unit increase**				
Crude model	1.65 (1.31–2.08)	<0.001	1.61 (1.29–2.01)	<0.001
Model 1	1.55 (1.23–1.97)	<0.001	1.57 (1.24–1.97)	<0.001
Model 2	1.31 (1.01–1.70)	0.046	1.35 (1.04–1.75)	0.024
Model 3	1.32 (0.99–1.74)	0.055	1.40 (1.06–1.85)	0.017

Crude model: unadjusted hazard ratios (HRs) and subdistribution hazard ratios (sHRs) for continuous METS-IR. HRs were estimated using Cox proportional hazards models, and sHRs were estimated using Fine–Gray subdistribution hazard models.

Model 1: METS-IR, age and sex.

Model 2: Model 1 plus medications and comorbidities.

Model 3: Model 2 plus PD-related parameters and laboratory data.

Abbreviations: METS-IR, metabolic score for insulin resistance; HR, hazard ratio; sHR, subdistribution hazard ratio; CI, confidence interval; SD, standard deviation.

### AUC and ROC curve analysis

The ROC curve was shown in Fig 2 and the exploratory cutoff value of METS-IR with area under the ROC curve was shown in Table 4. For the total population, the METS-IR cutoff point of 33.3 exhibited a SS of 75.3% and SP of 48.3%, with an AUC (95% CI) of 0.617 (0.557–0.677; p < 0.001) and a Youden-X of 0.238 for CV mortality.

**Table 4:** Calculation of the exploratory cutoff value of METS-IR for cardiovascular death using the area under the ROC curve.

	Criterion values and coordinates of ROC curve							Area under the ROC curve			
Index	Value	Sensitivity	Specificity	PPV	NPV	+LR	-LR	Area	SE	95% C.I.	P-value
METS-IR	>=33.3	0.753	0.485	0.219	0.911	1.462	0.510	0.617	0.030	0.557 −0.677	<0.001

ROC: Receiver operating characteristic.

Sensitivity: true positive rate; Specificity: true negative rate

LR + , positive likelihood ratio; LR − , negative likelihood ratio

LR+=Sensitivity/(1-Specificity); LR−=(1-Sensitivity)/Specificity

Area: area under the curve

The cut-off value was identified using the Youden index and should be interpreted as exploratory.

**Fig 2:**
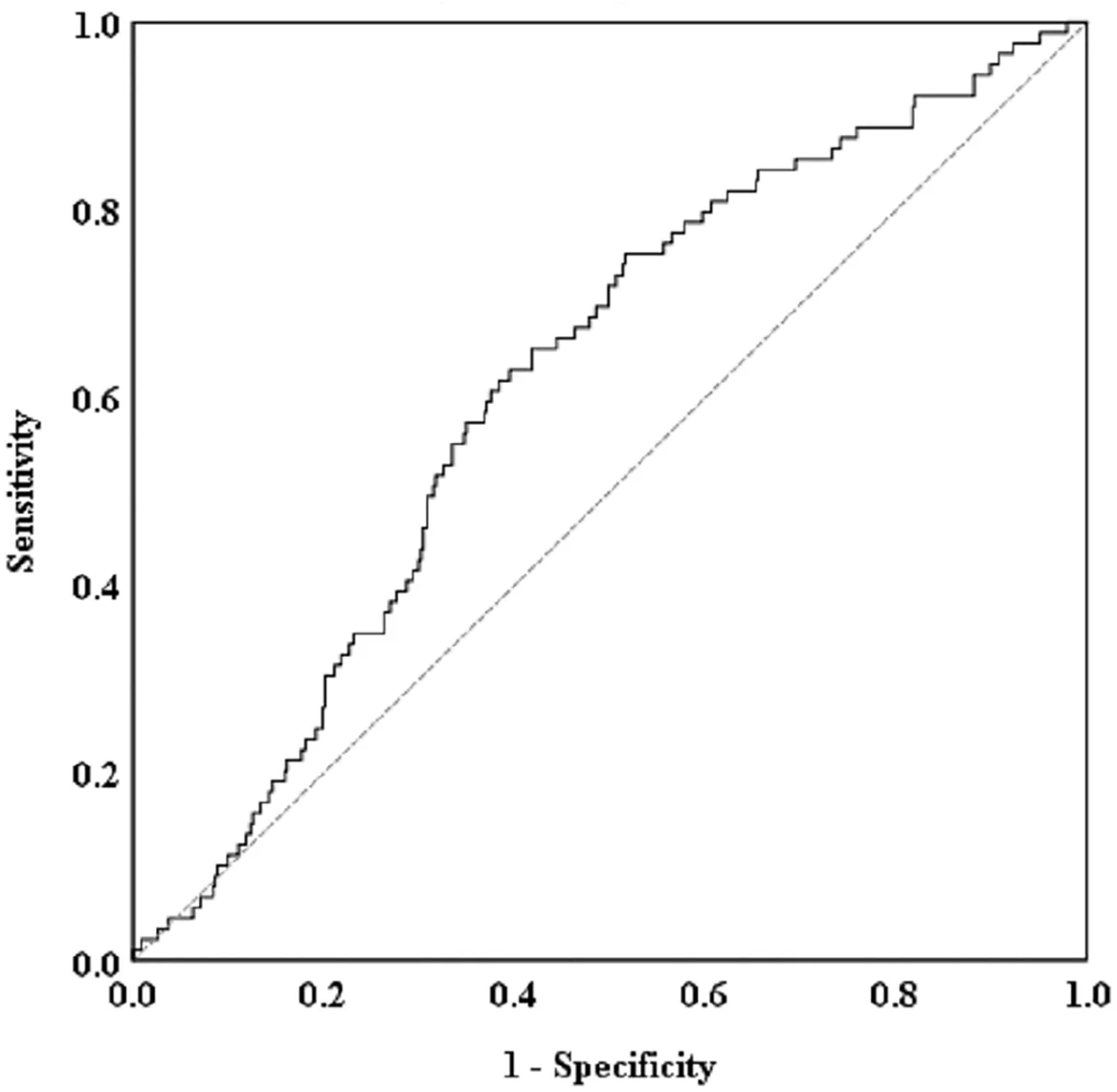
The area under the curve (AUC) by the receiver operating characteristics (ROC) curve analysis of the cardiovascular mortality for METS-IR.

### Sensitivity analysis

We further checked the independent role of METS-IR index for predicting CV mortality when using exploratory METS-IR threshold identified by ROC analysis (Table 2). In the fully adjusted model, patients with METS-IR ≧ 33.3 were associated with a HR of 1.89 (95% CI, 1.12–3.23; p = 0.018) compared with those with METS-IR < 33.3. Similar results were observed in the competing risk analysis using the Fine–Gray model (sHR 2.27, 95% CI 1.30–3.96; p = 0.004).

## Discussion

To the best of our knowledge, this is the first study assessing the association of METS-IR with CV mortality in ESRD patients on PD. Our study found that a higher METS-IR index was positively and independently associated with a higher risk of CV mortality both in the unadjusted and fully adjusted Cox proportional regression models. After adjusting all the confounding factors, patients in the METS-IR tertile 2 and tertile 3 were associated with HRs of 1.90 and 1.91 for CV mortality, respectively, with reference to tertile 1. Through ROC analysis, the exploratory METS-IR threshold value ≥33.3 was associated with an 89% higher risk of CV death. Furthermore, since non-CV deaths may constitute competing events when assessing CV mortality, we performed a competing risk analysis using the Fine-Gray model. The results were consistent with those of the Cox proportional hazards model, further supporting the robustness of our findings. Similar results were observed when METS-IR was analyzed as a continuous variable. Thus, our study suggests that METS-IR might serve as a useful and reliable biomarker to predict CV mortality among patients treated with PD. However, the discriminatory ability of METS-IR alone was modest in our study (AUC = 0.617), suggesting that METS-IR may be better considered as a marker of CV risk rather than a standalone predictive tool.

Also known as syndrome X, insulin resistance refers to a condition in which the body has reduced sensitivity and responsiveness to insulin. Hyperinsulinemia, caused by insulin resistance, can accelerate fatty acid production, hinders the normal action of insulin, and may result in CV changes, such as left ventricular remodeling and atherosclerosis in various detectable vascular areas [12]. It usually occurs several years before the onset of diabetes and long-term metabolic disorder has been shown to increase the risk of CV and all-cause mortality in patients with diabetes or pre-diabetes [13]. More than 50% of deaths in PD patients are attributed to CVD [14]. Despite significant advances in PD treatment, insulin resistance (IR) remains a key factor contributing to the extremely high mortality.

The long-term adverse consequences of metabolic derangement caused by insulin resistance will undoubtedly exacerbate hypertension, dyslipidemia, hyperglycemia and obesity, thereby leading to CV death. Several studies have reported the association of METS-IR with CVD in a variety of ethical populations. Using data from 28437 participants, a Koran study showed that compared with the referent first quartile, the highest METS-IR quartile significantly predicted future CVD with HR of 2.61 (95% CI 1.83–3.72), after adjusting for age, sex, and body mass index [15]. An Iran prospective study of community population reported METS-IR to be significantly associated with the risk of incident CHD, diabetes and hypertension [16]. A prospective cohort study in China by Wang et al. identified a significant link between METS-IR and CVD incidence in Xinjiang’s rural areas, concluding that regular METS-IR index monitoring can aid in the early detection of CVD-risk groups [17]. Later, a Chinses study involving community population with hypertension and without CVD observed a similar finding [18]. Moreover, a recent meta-analysis of cohort studies aimed to evaluate the relationship between baseline METS-IR and the incident of CVD and demonstrated that higher METS-IR was significantly correlated with higher risk of composite CVD, coronary artery disease and stroke [19]. These studies aligned with our findings suggesting METS-IR as an independent predictor of CV mortality.

Numerous studies on CKD or ESRD patients undergoing PD have shown that elevated triglyceride levels, impaired glucose metabolism, and increased BMI are associated with various metabolic disorders and mortality risk. Traditional glucose-containing PD solution is the first-line treatment option for most patients undergoing PD. However, long-term use of glucose-containing PD solution can adversely affect mortality, especially CV mortality [20]. Recent clinical studies have highlighted the relationship between insulin resistance and CVD progression in CKD patients. “Uremic dyslipidemia” is a lipoprotein pattern specific to patients with chronic kidney disease and high levels of triglycerides can promote inflammation, oxidative stress, endothelial dysfunction, and other characteristics of atherosclerosis [21]. J.A. et al. showed that high TG levels were associated with increased risk of atherosclerotic vascular events in CKD [22]. Furthermore, FBG is an independent predictor of CVD-free survival in patients receiving PD [23]. In patients undergoing PD, a higher BMI is strongly associated with an increased risk of CV death [24]. In addition, epidemiological studies have shown that low levels of high-density lipoprotein cholesterol are a risk factor for CVD in the general population [25,26]. The above findings indicate that all components of METS-IR (TG, FBG, HDL-C, and BMI) are associated with patients’ clinical outcomes. In the present study, METS-IR was independently associated with CVD mortality in our PD cohort.

The reasons why higher METS-IR increases CV mortality could be proposed by the biological mechanisms associated with insulin resistance or hyperinsulinemia. While considering CVD, IR can lead to a number of cardiovascular dysfunction and structural abnormalities via endothelial and myocardial damage through several interconnected pathways [6]. In addition to traditional metabolic tissues (liver, skeletal muscles and adipocytes), IR also exert its deleterious effects on CV cells [27]. Normally, insulin exerts its protective effect on CV systems. In pathological conditions, hyperinsulinemia can lead to an imbalance in glucose homeostasis, resulting in chronic hyperglycemia, which in turn promotes oxidative stress and activates pro-inflammatory pathways, such as NF-κB. Aberrant insulin signaling can also alter systemic lipid metabolism, induce lipotoxicity, impair nitric oxide production and foster atherosclerosis and increase susceptibility to myocardial ischemia [7,28,29]. In addition, vascular insulin resistance impairs the selective activation of the PI3K/NO pathway and compensatory hyperinsulinemia activates the MAPK pathway, leading to vascular smooth muscle cell proliferation, vascular hypertrophy, vascular stiffness, and promoting the development of CVD [30]. These pathophysiological processes form the molecular basis for the association between elevated METS-IR, a surrogate marker of insulin resistance. and increased risk of CV death.

Previous studies have shown that insulin resistance, measured by indirect/surrogate indicators, is associated with parameters of metabolic disturbance and adverse clinical outcomes in patients undergoing PD. For example, a Korean PD study found that homeostatic model assessment of insulin resistance (HOMA-IR) was associated with an increased risk of new major CV events, with the highest tertile group having a 1.18-fold higher risk than the lowest tertile group [31]. Furthermore, a Chinese PD cohort study showed that PD patients with a TyG index >8.69 had almost three times the risk of CV mortality compared to those with a TyG index <8.10 [32]. Recently, Wan et al. reported similar results, showing that patients with the highest TyG index had a significantly higher risk of CV mortality than those in the lowest tertile group (HR, 4.48, 95% CI 1.51–13.56) [33]. In addition, a study involving 2,335 PD patients also demonstrated that TyG-BMI was associated with an increased risk of CV mortality [34]. Consistent with previous findings on insulin resistance and CV outcomes, we also observed that METS-IR was significantly associated with the risk of CV mortality after adjusting for essential confounders among our incident PD patients.

This PD-based cohort study has several limitations that need to be addressed. First, the retrospective nature of this study limits causal inferences. Association is not the same as causation, as many confounding factors, such as genetic predisposition and lifestyle influences, may play a key role in mediating such associations. Second, establishment of the normal value, cut-off point, and high-risk range of METS-IR in PD patients would greatly enhance its clinical applicability in predicting CV mortality. Due to the lack of baseline insulin level data, we cannot either compare METS-IR with HOMA or establish the normal ranges specific to PD patients. Fourth, given that this is an observational study, we should be aware of potential residual confounding factors. Finally, since this is a single-center study in Taiwan, the results may not be generalized to other countries. Finally, the ROC derived thresholds determined in this study should be interpreted with caution, as it was derived from the same dataset and has not been externally validated.

In conclusion, METS-IR is reportedly a cost-effective and easy-to-use scoring system based on routine available clinical parameters, therefore it emerges as an innovative alternative index to assess IR. According to our research, METS-IR can identify PD patients at high risk of CV death. However, a modest adjusted HR of 1.91 and an AUC of only 0.617 indicated limited discriminative ability and only moderate predictive ability. Despite its limitations, our novel findings support the use of METS-IR to assess the risk of CV death in patients receiving PD therapy, which means that patients with high METS-IR values should receive more medical attention to improve CV survival.
